# Increased AURKA promotes cell proliferation and predicts poor prognosis in bladder cancer

**DOI:** 10.1186/s12918-018-0634-2

**Published:** 2018-12-14

**Authors:** Mengjie Guo, Sicheng Lu, Hongming Huang, Yaohui Wang, Mary Q. Yang, Ye Yang, Zhimin Fan, Bin Jiang, Youping Deng

**Affiliations:** 1 School of Medicine and Life Sciences, Nanjing University of Chinese Medicine, Nanjing, 210023 China; 2 Department of Hematology, Affiliated Hospital of Nantong University, Nantong, 226001 China; 3 Department of Pathology, Jiangsu Province Hospital of Traditional Chinese Medicine, Nanjing, 210029 China; 4 MidSouth Bioinformatics Center, Department of Information Science, George Washington Donaghey College of Engineering and Information Technology and Joint Bioinformatics Graduate Program, University of Arkansas at Little Rock and University of Arkansas for Medical Sciences, Little Rock, AR 72204 USA; 5 National Medical Centre of Colorectal Disease, The Third Affiliated Hospital of Nanjing University of Chinese Medicine, Nanjing, 210001 China; 6 Integrated Medical College, Nanjing University of Chinese Medicine, Nanjing, 210023 China; 7 Bioinformatics Core, Department of Complementary & Integrative Medicine, University of Hawaii John A. Burns School of Medicine, Honolulu, HI 96813 USA

**Keywords:** Bladder cancer, Aurora kinase a (AURKA), Oncogene, Apoptosis

## Abstract

**Background:**

Bladder cancer (BC) is the most common cancer of the urinary bladder and upper tract, in which the clinical management is limited. AURKA (aurora kinase A) has been identified as an oncogene in cancer development; however, its potential role and underlying mechanisms in the progression of BC remain unknown.

**Results:**

In this study, we evaluated Aurora kinase A (AURKA) expression in patient samples by performing gene expression profiling, and found that AURKA expression levels were significantly higher in BC tissues than in normal tissues. Increased AURKA in BC was strongly associated with stage and grade. Moreover, BC patients with elevated AURKA achieved poor overall survival rates. The experiments *in vitro* comprehensively validated the critical role of AURKA in promoting BC cell proliferation using the methods of gene overexpression and gene silencing. Furthermore, we proved that AURKA inhibitor MLN8237 arrested BC cell growth and induced apoptosis.

**Conclusions:**

These findings implicate AURKA acting as an effective biomarker for BC detection and prognosis, as well as therapeutic target.

## Background

Bladder cancer (BC) is the most prevalent malignant tumor of the urinary system, ranked as 9th commonest cause of cancer worldwide [1]. Irrespective of significant improvements in management, the recurrence and progression rates are still high, with a marked upward trend of morbidity and mortality annually [2]. Among the initial diagnosis, approximately 70% of patients are with non-muscle-invasive (non-MI) disease (stages Ta/T1/Tis), while others are muscle-invasive (MI) (stages T2-T4,[3]). Unfortunately, 20–30% of patients with non-MI tumors will eventually progress to a higher grade or stage such as MI disease during surveillance [4]. The prognosis for patients with advanced disease remains poor: 5-year survival rates are around 20% or lower for surgically incurable patients [5]. Clinicopathological factors such as stage and grade have been served as the substantial predictors of outcome, which are associated with enhanced possibilities of progression [6]. Many clinical trials have shown that BC is driven by genetic variations resulting in uncontrolled biologic behavior of the tumor [7]. Consequently, it is urgent to find novel molecular markers providing invaluable clues related to pathogenesis and prognosis in BC.

Aurora kinase A (AURKA) is a crucial member of the Aurora/Ipl1p kinase family, acting as a cell-cycle associated kinase on maintaining genomic integrity [8, 9]. To ensure the successful completion of mitosis, AURKA is involved in separation and maturation of the central body, as well as stabilization at the spindle pole during chromosome segregation [10]. Aberrant activity of AURKA facilitates tumorigenic transformation and progression through defective control of the mitotic spindle checkpoint in mammalian cells and in several types of human tumors, including breast cancer [11], colorectal cancer [8], myeloid leukemia [12] and hepatocellular carcinoma [13]. The above points make AURKA as a tumor susceptibility locus [14, 15].

In this study, we performed sequential gene expression profiling (GEP) from patients of primary bladder cancer (PBC) or undergoing radical cystectomy (RC) to reveal the relationship between AURKA expression, clinical characteristics and overall survival. Finally, we aim to underlie the mechanism of AURKA in the progression of BC.

## Results

### AURKA is overexpressed in BC tissues

**Fig. 1:**
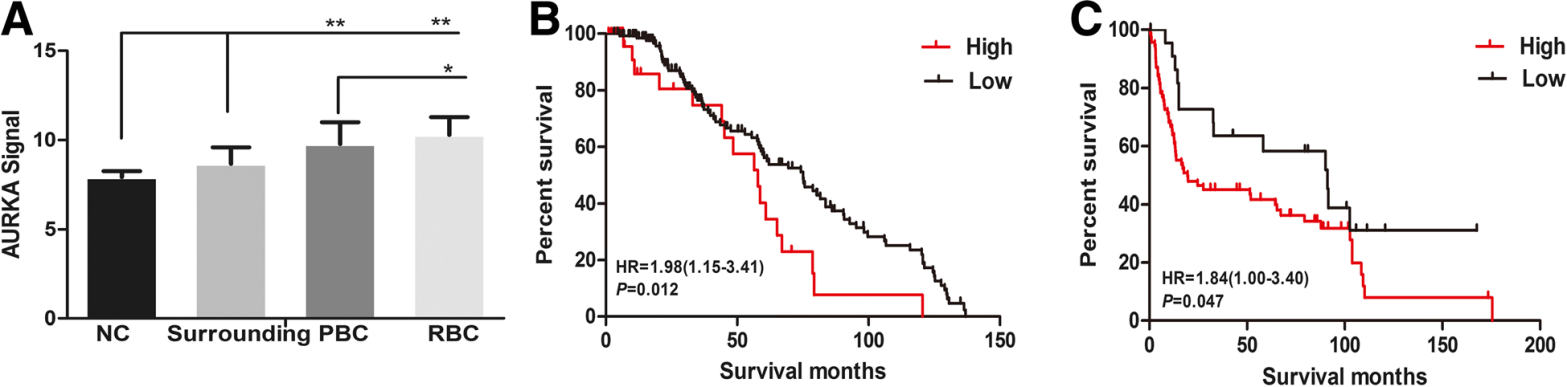
AURKA levels are correlated with diagnosis and poor survival in bladder cancer patients. **a** AURKA expression of normal control cells (NC, *n* = 10), normal looking bladder mucosae with surrounding carcinoma (Surrounding, *n* = 58), primary bladder cancer (PBC, *n* = 165) and recurrent bladder cancer (RBC, *n* = 23) in GEP dataset. **b** and **c** Kaplan-Meier analysis on the overall survival of bladder cancer patients in **b** GSE13507 and **c** GSE31684 dataset based on the AURKA expression. (^*^P < 0.05, ^**^P < 0.01)

We explored the expression of AURKA in NC, Surrounding, PBC and RBC samples by performing GEP database analysis. AURKA expression was remarkably increased in PBC and RBC cells compared to NC and Surrounding (*P* = 0.000) (Fig. 1a). Furthermore, the outcome presented that AURKA expression was extremely higher in RBC samples than in PBC samples (*P* = 0.043).

### Increased AURKA is associated with clinicopathologic characteristics in BC patients

The expression level of AURKA was extracted and correlated with available clinical parameters for the database of GSE13507 and GSE31684 (Table 1 and 2). In GSE13507, high expression of AURKA in PBC was dramatically associated with tumor stage (χ^2^ = 11.815, *P* = 0.019), grade (χ^2^ = 17.927, *P* = 0.000), invasiveness (χ^2^ = 13.229, *P* = 0.001), and cancer-specific survival (χ^2^ = 4.557, *P* = 0.042). Similarly, high expression of AURKA in GSE31684 was closely linked to characteristics like stage (χ^2^ = 18.766, *P* = 0.001) and grade (χ^2^ = 11.833, *P* = 0.003). Nevertheless, no significant correlations between AURKA levels and gender, age, histology type, lymph node status and metastasis were observed in both datasets. The above results suggested that AURKA may play a role as potential diagnosed marker in BC.

**Table 1:** Correlation of AURKA expression and clinicopathologic characteristics of PBC in the datasheet of GSE 13507

Characteristic	n	AURKA expression (%)		Pearson χ^2^	*P*
		High	Low or no		
Total	165	30 (18.18)	135 (81.82)		
Age				3.460	0.069
≥ 65	96	22 (22.92)	74 (77.08)		
< 65	69	8 (11.59)	61 (88.41)		
Gender				1.774	0.196
Female	30	8 (26.67)	22 (73.33)		
Male	135	22 (16.30)	113 (83.70)		
Tumor stage				11.815	0.019*
Ta	24	3 (12.50)	21 (87.50)		
T1	80	8 (10.00)	72 (90.00)		
T2a + T2b	31	11 (35.48)	20 (64.52)		
T3a + T3b	19	5 (26.31)	14 (73.69)		
T4a + T4b	11	3 (27.27)	8 (72.73)		
Grade				17.927	0.000**
low	105	9 (8.57)	96 (91.43)		
high	60	21 (35.00)	39 (65.00)		
Invasiveness				13.229	0.001**
superical	103	10 (9.71)	93 (90.29)		
invasive	62	20 (32.26)	42 (67.74)		
Systemic chemo				0.002	1.000
Yes	27	5 (18.52)	22 (81.48)		
No	138	25 (18.11)	113 (81.89)		
Lymph node status				5.035	0.184
Nx	1	0 (0.00)	1 (100.00)		
N0	149	26 (17.45)	123 (82.55)		
N1	8	2 (25.00)	6 (75.00)		
N2	6	1 (16.67)	5 (83.33)		
N3	1	1 (100.00)	0 (0.00)		
Progression				1.492	0.299
No	134	22 (16.42)	112 (83.58)		
Yes	31	8 (25.81)	23 (74.19)		
Metastasis				0.075	1.000
No	158	29 (18.35)	129 (81.65)		
Yes	7	1 (14.28)	6 (85.72)		
Cancer-specific survival				4.557	0.042*
Survival	133	20 (15.04)	113 (84.96)		
Death	32	10 (31.25)	22 (68.75)		

^*^P<0.05, ^**^P<0.01 significant difference between clinicopathologic characteristics of patients with high and low AURKA expression

**Table 2:** Correlation of AURKA expression and clinicopathologic characteristics in the datasheet of GSE31684

Characteristic	n	AURKA expression (%)		Pearson χ^2^	*P*
		High	Low or no		
Total	93	70 (75.27)	23 (24.73)		
Age				2.596	0.122
≥ 65	65	52 (80.00)	13 (20.00)		
< 65	28	18 (64.29)	10 (35.71)		
Gender				0.010	1.000
Female	25	19 (76.00)	6 (24.00)		
Male	68	51 (75.00)	17 (25.00)		
RC stage				18.766	0.001**
pTa	5	0 (0.00)	5 (100.00)		
pT1	10	6 (60.00)	4 (40.00)		
pT2	17	13 (76.47)	4 (23.53)		
pT3	42	35 (83.33)	7 (16.67)		
pT4	19	16 (84.21)	3 (15.79)		
RC grade				11.833	0.003**
high	87	69 (78.16)	18 (21.84)		
low	6	1 (16.67)	5 (83.33)		
Histology				2.487	0.288
TCC	86	63 (73.25)	23 (26.75)		
TCC/CIS	2	2 (100.00)	0 (0.00)		
TCC/square	5	5 (100.00)	0 (0.00)		
Lymph node status				5.876	0.053
PN0	49	32 (65.31)	17 (34.69)		
PN+	28	25 (89.28)	3 (10.72)		
PNx	16	13 (81.25)	3 (18.75)		
Recurrence				0.304	0.635
No	52	38 (73.08)	14(26.92)		
Yes	41	32 (78.05)	9 (21.95)		
Last known status				2.651	0.266
NED	28	18 (64.28)	10 (35.72)		
DOC	27	22 (81.48)	5 (18.52)		
DOD	38	30 (78.95)	8 (21.05)		
Metastasis				0.199	0.806
No	57	42 (73.68)	15 (26.32)		
Yes	36	28 (77.78)	8 (22.22)		

^**^P<0.01 significant difference between clinicopathologic characteristics of patients with high and low AURKA expression

### High AURKA levels are correlated to poor survival

As described in (Fig. 1b and c), kaplan-Meier survival curves showed a connection between high expression of AURKA in the primary tumors and reduced overall survival time of patients (HR = 1.98, *P* = 0.012). Simultaneously, higher AURKA expression was linked to considerably shorter response duration of survival (HR = 1.84, *P* = 0.047). It indicated that AURKA may act as a prognostic marker in BC.

### AURKA overexpression forces the proliferation of BC cells

**Fig. 2:**
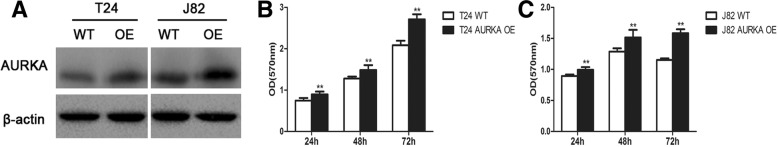
AURKA overexpression forces the proliferation of BC cells. **a** AURKA expression in T24 and J82 cells were detected by western blot after AURKA-cDNA transfection. **b** and **c** Cell proliferation of **b** T24 and **c** J82 AURKA-overexpression (OE) and untransfected cells (WT). (^*^P < 0.05, ^**^P < 0.01)

To further verify that AURKA is a driver for BC cell proliferation, we overexpressed AURKA in two cell lines (T24 and J82). The elevated expression of AURKA in the overexpressing cells (OE) compared to the untransfected control cells (WT) was validated by Western blot (Fig. 2a). We evaluated the differences of growth rate between WT and OE cells in short-term cultures for three time points. The result showed that the cells exhibited a significantly increased rate of proliferation in OE cells relative to WT cells (*P* = 0.000) (Fig. 2b), which point out that AURKA is required for the promotion of BC cell growth *in vitro*.

### Silencing AURKA expression inhibits the proliferation of BC cells

**Fig. 3:**
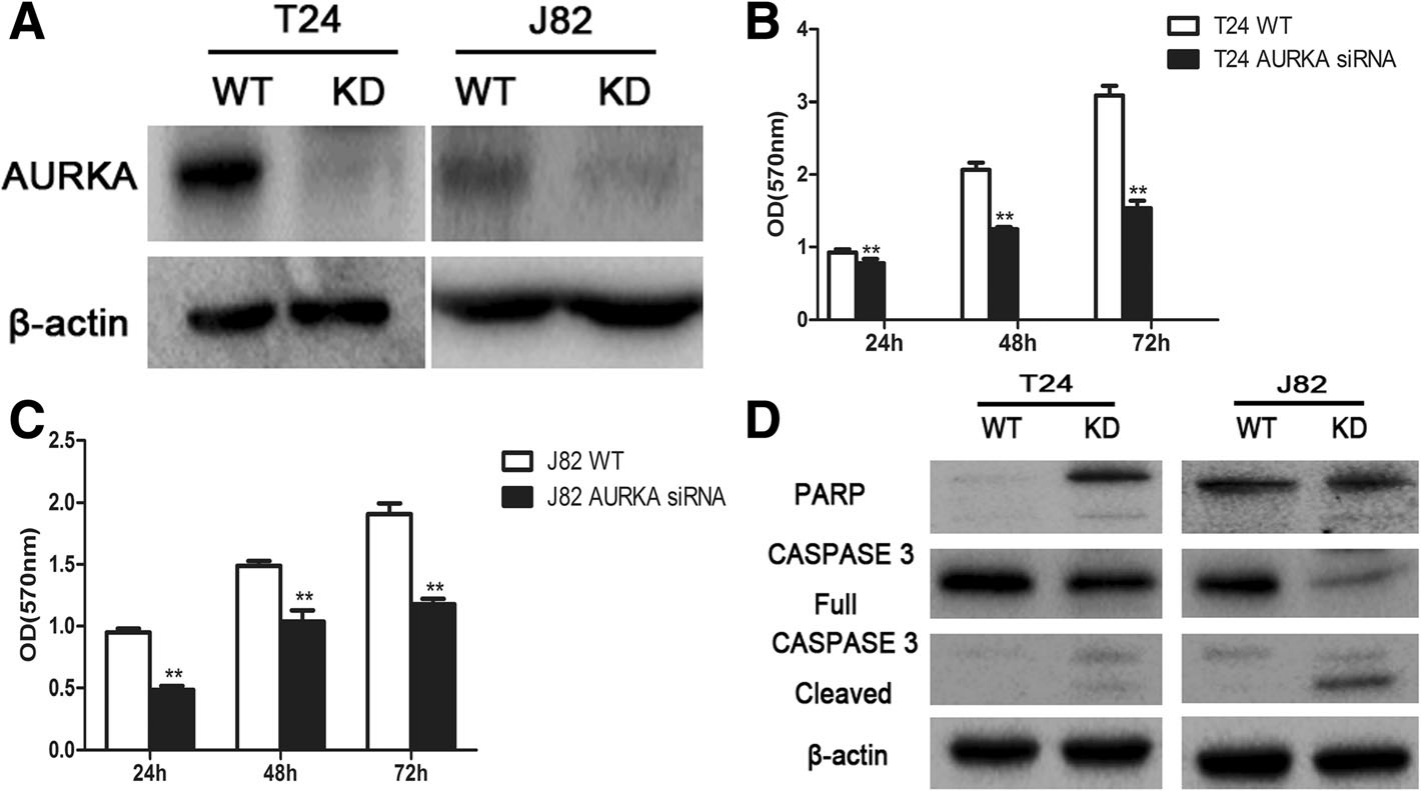
Silencing AURKA expression inhibits the proliferation of BC cells. **a** AURKA expression in T24 and J82 cells were detected by western blot after AURKA-siRNA transfection. **b** and **c** Cell proliferation of **b** T24 and **c** J82 AURKA-knockdown (KD) and untransfected cells (WT). **d** Western blot of T24 and J82 Ctrl and KD cells on the PARP and Caspase 3 expression. (^*^P < 0.05, ^**^P < 0.01)

siRNA technology was utilized to knockdown the endogenous expression of the AURKA gene in BC cells, which was confirmed by western blot as well. As shown in (Fig. 3a), AURKA expression was markedly reduced in BC cells transfected with AURKA siRNA (KD) compared to the control (WT). As shown in (Fig. 3b and c), the proliferation rate of T24 and J82 cells was drastically decreased followed by silencing AURKA at the three time points (*P* = 0.000). Subsequently, cleaved PARP and Caspase-3 proteins were examined by western blotting to explore the functional mechanism of AURKA. Under the circumstance of siRNA interference, the protein levels of cleaved PARP and Caspase-3 indicated the induction of apoptosis. Thus, the results further reinforce that AURKA displays an impressively positive effect on proliferation of BC cells *in vitro*.

### MLN8237 treatment suppresses BC cell proliferation

**Fig. 4:**
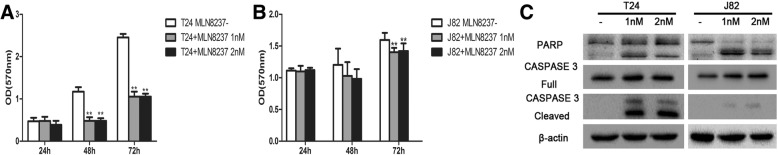
MLN8237 treatment suppresses BC cell proliferation. **a** and **b** Cell proliferation of **a** T24 and **b** J82 cells after MLN8237 treatment. **c** PARP and Caspase 3 expression by Western blot in T24 and J82 cells after MLN8237 treatment (^*^P < 0.05, ^**^P < 0.01)

We employed the AURKA selective inhibitor MLN8237 to evaluate its antitumor activity via targeting AURKA on the T24 and J82 cell lines. Treatment with 1 nM and 2 nM MLN8237 respectively in T24 and J82 cells for 72 h exerted apparent inhibiton to BC cell growth (*P* < 0.01). The proliferation of T24 and J82 cells from initiation of treatment to 24 h remained unaltered. The 48-h treatment exhibited significant impact on inhibiting the proliferation of T24 cell (*P* < 0.01), as well as the growth of J82 cells was suppressed to a certain extent (Fig. 4a and b). Western blot analysis to test PARP and Caspase-3 in T24 and J82 cells revealed that cleaved PARP and Caspase-3 proteins levels were highly increased (Fig. 4c). Taken together, the results underscored the potential of MLN8237 as an effective agent targeting AURKA in BC cells.

## Discussion

Several observations have found that chromosomal anomalies, genetic polymorphisms, genetic and epigenetic alterations are involved in the tumorigenesis and progression of bladder cancer [16, 17]. Human chromosome 20 is of special interest due to its crucial role in the pathogenesis of diverse cancers [18, 19]. Gains and amplifications at chromosome 20q13.2 resulted in the induction and transformation of primary human urothelial cells, suggesting that overexpression of a gene or genes at this chromosomal locus is linked with the bladder tumor aggressiveness [20]. As a result, most of severe dysplasia or carcinoma manifests a high tendency to develop into high-grade papillary and nonpapillary tumors [15]. Thus, there is a dire need to investigate the specific oncogenes and gene amplification events at chromosome 20, especially at the 20q13 region in BC.

Accumulating evidence with respect to the studies of oncogene has proved that AURKA, belonging to a family of mitotic kinases that maintain chromosomal stability, could promote tumor cell proliferation, diving tumorigenesis and tumor progression [21–23]. Amplification of AURKA has been observed in primary human urothelial cells at chromosome 20q13.2, which may play a key role in the development of BC [20]. In addition, overexpression of AURKA is interrelated with higher stages, grades and worse survival [24]. Zhou et al. showed that treating with AUKRA inhibitor increases the expression of apoptotic markers such as cleaved poly ADP-ribose polymerase (PARP) precipitating the suppression of BC cell proliferation [5]. Park et al. first investigated AURKA gene copy number in exfoliated cells of voided urine samples, and found that the sensitivity, specificity and area under the ROC curve were 96.6, 87 and 93.9%, respectively [15]. Our research combined gene expression omnibus data excavation, meta-analysis and *in vitro* experiments to obtain an exhaustive estimation of AURKA expression in the carcinogenesis and progression of BC.

Significant elevated AURKA was observed in PBC and RBC cells compared to NC and Surroundings by using publicly available GEP datasets. Stage and grade have been regarded as one of the most critical predictors of tumor recurrence and progression [25]. Our findings are consistent with the previous reports that higher expression of AURKA is associated with higher grades and stages [26, 27]. More importantly, patients with high AURKA expression suffered from worse survival than AURKA low-expressing patients. Anti-apoptosis was regarded as a distinct characteristic of tumor development [28]. Previous research has revealed the positive regulatory function of cleaved PARP and Caspase-3 in the onset of apoptosis [29]. Activation of the apoptotic pathway following AURKA inhibition constitutes an important mechanism for achieving cell death [30]. Our data was supported by the other findings that transfection of miR-124-3p mimics and AURKA siRNA down-regulate BC cell proliferation and migration as well as induce cell apoptosis [9]. In addition, MLN8237 actives cleaved PARP and Caspase-3 resulting in cell apoptosis, which is in accordance with the previous research performing on esophageal adenocarcinoma cells [31]. MLN8237 has been tested in various phase I and phase II clinical trials for advanced solid tumors and hematologic malignancies [32]. These data provide useful information for further therapeutic investigation of MLN8237 and other AURKA inhibitors in BC.

## Conclusions

Collectively, our findings have disclosed the oncogenetic functions of AURKA in BC based on its prospective diagnostic and prognostic significance. Targeting AURKA may afford a novel treatment approach to bladder cancer.

## Methods

### Cell lines and cell culture

Human BC cell lines, T24 and J82, were maintained in DMEM and α-MEM medium ((Hyclone Laboratory, Logan, UT), respectively, both of which were supplemented with 10% heat-inactivated fetal bovine serum (FBS) (Gibco, Grand Island, NY), 1% penicillin and streptomycin (P/S) solution (Sigma, St. Louis, MO) at 37 °C in a 5% CO_2_ incubator.

### Reagents

Antibodies of AURKA, Caspase-3 and cleaved PARP were purchased from Cell Signaling Technology (Danvers, MA). GAPDH antibody was obtained from SinoBiological (Beijing, China). AURKA inhibitor, MLN8237, was supplied by Aladdin (Shanghai, China), with the relative molecular weight of 518.94. MLN8237 stock solution (8 μM) was prepared in DMSO and diluted into 1 nM and 2 nM with cell culture media.

### Cell transfection

#### Plasmid transfection

The plasmid for overexpression of AURKA was constructed as previous described [33], named as pCMV2-FLAG-AURKA. Its reference sequence is NM_198433.1. The size is 1212 bp in total, of which the position ranging from 567 to 1778. T24 and J82 cells were incubated at 37 °C and 5% CO_2_ overnight until the cells had reached 80%~ 90% confluences. 1 μg of pCMV2-FLAG-AURKA and 2 μL of lip2000 were added to T24 and J82 cells with penicillin and streptomycin-free medium. Cells were incubated at 37 °C and 5% CO_2_ for 6 h, the medium was then replaced with complete medium. Transfected efficiency was verified by Western blot.

### RNA interference

The AURKA-siRNA sequence was as following: AURKA-Homo-947 5′~ 3’:GGGCUUUGGAAGACUUUGATTUCAAAGUCUUCCAAAGCCCTT. Sequences were chemically synthesized by GenePharma Co., Ltd. (Shanghai, China). For transfection experiments with siRNA directed against AURKA, the medium for T24 and J82 cells were changed to penicillin and streptomycin-free medium and confluence of cells were 30–50%. Then, the cells were cotransfected with 2 μL of siRNA and 1 μL of lip2000. After 6 h, the medium was changed to complete medium. Transfected efficiency was verified by Western blot.

### Cell growth assays

Following transfection or MLN8237 treament, cell growth was evaluated using an MTT assay at 24 h, 48 h and 72 h after seeding. Cells were inoculated onto 96-well plates at the density of 4000~ 5000 cells/well. After incubated for 2 h–4 h, two concentrations of MLN8237 were added to the cells. After 48 h continuous exposure to the compounds at 37 °C and 5% CO_2_, 10 μL of MTT and 90 μL of complete medium were added to each well, incubating for 4 h. The culture medium was removed and 150 μL dimethyl sulfoxide was added to each well. After vigorous shaking for 10 min, the absorbance (optical density, OD) of the reaction solution was measured at 570 nm by microplate reader.

### Western blot analysis

Western blots were utilized to measure the protein levels of AURKA and apoptotic markers in BC cells. In brief, around 20 μg protein per sample was extracted and analyzed by SDS-PAGE. Then the cell extract was transferred to a 0.45 μm immobilon-P transfer membrane (Millipore, Bedford, MA). After incubated with primary and second antibodies, detection of specific proteins was carried out by ECL (Amersham Pharmacia Biotech, Piscataway, NJ).

### Gene expression profiling (GEP) and data analysis

Gene expression data were publicly available from NIH Gene Expression Omnibus. The datasets of bladder cancer used in our study were GSE13507 (*n* = 256) and GSE31684 (*n* = 93). The GSE13507 dataset contains 10 normal bladder mucosae (NC), 58 normal looking bladder mucosae with surrounding carcinoma (Surrounding), 165 primary bladder cancer (PBC) samples and 23 recurrent bladder cancer (RBC) samples. The GSE31684 dataset contains 93 patients undergoing radical cystectomy (RC). GEP was conducted by Affymetrix U133 Plus 2.0 microarrays as previously described [34, 35].

### Statistical analysis

All data were presented as means ± SD. Two experimental groups were analyzed by Student’s t test, while multiple (*n* ≥ 3) groups were analyzed with one-way ANOVA. The patient survival data were sketched by the Kaplan-Meier method, and a log-rank test was performed to estimate survival curves. A Chi-square test was performed for clinicopathologic categorical variables to compare the high expression cohort with the low expression cohort. The significance level was set at *P* < 0.05.

## Abbreviations

AURKA: Aurora kinase A
BC: Bladder cancer
GEP: Gene expression profiling
MI: Muscle-invasive
PARP: Poly ADP-ribose polymerase
PBC: Primary bladder cancer
RBC: Recurrent bladder cancer
RC: Radical cystectomy
